# Altered aortic 3D hemodynamics and geometry in pediatric Marfan syndrome patients

**DOI:** 10.1186/s12968-017-0345-7

**Published:** 2017-03-17

**Authors:** Roel L. F. van der Palen, Alex J. Barker, Emilie Bollache, Julio Garcia, Michael J. Rose, Pim van Ooij, Luciana T. Young, Arno A. W. Roest, Michael Markl, Joshua D. Robinson, Cynthia K. Rigsby

**Affiliations:** 1 Department of Radiology, Feinberg School of Medicine, Northwestern University , Chicago, IL USA; 2 Division of Pediatric Cardiology, Department of Pediatrics, Leiden University Medical Center, Albinusdreef 2, 2333 ZA Leiden, The Netherlands; 3 Department of Cardiac Sciences, Stephenson Cardiac Imaging Centre, University of Calgary - Cumming School of Medicine, Calgary, AB Canada; 4 Department of Medical Imaging, Ann & Robert H. Lurie Children’s Hospital of Chicago, Chicago, IL USA; 5 Department of Radiology, Academic Medical Center, Amsterdam, The Netherlands; 6 Department of Biomedical Engineering, McCormick School; of Engineering, Northwestern University, Chicago, IL USA; 7 Department of Pediatrics, Division of Pediatric Cardiology, Ann & Robert H. Lurie Children’s Hospital of Chicago, Chicago, IL USA; 8 Department of Pediatrics, Feinberg School of Medicine, Northwestern University, Chicago, IL USA

**Keywords:** Marfan syndrome, Aorta hemodynamics, Aortic geometry, 4D flow, Children

## Abstract

**Background:**

Blood flow dynamics make it possible to better understand the development of aortopathy and cardiovascular events in patients with Marfan syndrome (MFS). Aortic 3D blood flow characteristics were investigated in relation to aortic geometry in children and adolescents with MFS.

**Methods:**

Twenty-five MFS patients (age 15.6 ± 4.0 years; 11 females) and 21 healthy controls (age 16.0 ± 2.6 years; 12 females) underwent magnetic resonance angiography and 4D flow CMR for assessment of thoracic aortic size and 3D blood flow velocities. Data analysis included calculation of aortic diameter and BSA-indexed aortic dimensions (Z-score) along the thoracic aorta, 3D mean systolic wall shear stress (WSS_mean_) in ten aortic segments and assessment of aortic blood flow patterns.

**Results:**

Aortic root (root), ascending (AAo) and descending (DAo) aortic size was significantly larger in MFS patients than healthy controls (Root Z-score: 3.56 ± 1.45 vs 0.49 ± 0.78, *p* < 0.001; AAo Z-score 0.21 ± 0.95 vs −0.54 ± 0.64, *p* = 0.004; proximal DAo Z-score 2.02 ± 1.60 vs 0.56 ± 0.66, *p* < 0.001). A regional variation in prevalence and severity of flow patterns (vortex and helix flow patterns) was observed, with the aortic root and the proximal DAo (pDAo) being more frequently affected in MFS. MFS patients had significantly reduced WSS_mean_ in the proximal AAo (pAAo) outer segment (0.65 ± 0.12 vs. 0.73 ± 0.14 Pa, *p* = 0.029) and pDAo inner segment (0.74 ± 0.17 vs. 0.87 ± 0.21 Pa, *p* = 0.021), as well as higher WSS_mean_ in the inner segment of the distal AAo (0.94 ± 0.14 vs. 0.84 ± 0.15 Pa, *p* = 0.036) compared to healthy subjects. An inverse relationship existed between pDAo WSS_mean_ and both pDAo diameter (*R* = −0.53, *p* < 0.001) and % diameter change along the pDAo segment (*R* = −0.64, *p* < 0.001).

**Conclusions:**

MFS children and young adults have altered aortic flow patterns and differences in aortic WSS that were most pronounced in the pAAo and pDAo, segments where aortic dissection or rupture often originate. The presence of vortex flow patterns and abnormal WSS correlated with regional size of the pDAo and are potentially valuable additional markers of disease severity.

**Electronic supplementary material:**

The online version of this article (doi:10.1186/s12968-017-0345-7) contains supplementary material, which is available to authorized users.

## Background

Marfan syndrome (MFS) is an inherited autosomal dominant connective tissue disease, mostly related to mutations in the fibrillin-1 (FBN1) gene. Many organ systems can be involved, but most life-threatening complications are related to the cardiovascular system and include aortic dissection and aortic rupture. Although the entire aorta may dilate in MFS, specific aortic regions are prone for progressive dilation and dissection and represent the aortic root and the proximal descending aorta (pDAo) [1–3]. Before the implementation of preventive surgical management strategies, two-thirds of dissections and ruptures occurred in the ascending aorta (AAo) while one-third occurred in the descending aorta (DAo). Recently, this ratio has shifted towards proportionally more DAo complications [4, 5]. Thus, pathology of the aorta distal to the aortic root remains a cause of concern.

In the current guidelines, absolute aortic diameters and identification of changes in aortic dimension play an important role in risk stratification and decision making for preventive surgery [6]. However, it is known that aortic dissection may occur in only moderately dilated aortas, and recent literature has shown that only about 50% of MFS patients with pDAo dissections had pDAo diameters larger than the upper limit of normal [5]. These findings indicate that aortic dimension alone may not capture the predictive risk for adverse cardiovascular events.

It is not well known how aortic hemodynamics interact with the altered vascular structure of the aorta in MFS. Independent associations between hemodynamic markers and aortopathy have been presented in cardiovascular magnetic resonance (CMR) imaging studies [7–10]. These studies employed time-resolved 3D phase-contrast CMR with three-directional velocity encoding (4D flow CMR) to enable a comprehensive, non-invasive in-vivo investigation of cardiac and aortic hemodynamics. The technique allows for quantitative analysis of advanced hemodynamic parameters [11–14] such as wall shear stress (WSS), which is defined as the tangential force exerted by blood flow on the aortic wall. WSS has recently been correlated with evidence of molecular and architectural medial wall dysfunction in bicuspid aortic valve-related aortopathy [15]. WSS may also be a useful parameter to identify focal regions with wall dysfunction in MFS patients. The aim of the study was to investigate regional aortic WSS, flow patterns and aortic dimensions in a pediatric MFS cohort compared to a healthy age appropriate control cohort by using a novel 3D WSS technique.

## Methods

### Study population

Twenty-five patients with MFS (mean age 15.6 ± 4.0 years, 11 females) were included in this Institutional Review Board (IRB) approved and HIPAA compliant study. All patients underwent 4D flow CMR immediately following a clinically ordered standard-of-care CMR assessment of the entire aorta. The diagnosis of MFS was based on clinical criteria according to the international standards derived from the 2010 Revised Ghent Criteria [16]. In addition to the clinical criteria, conventional genetic testing for FBN1 mutation was performed in 23 MFS patients; in 22 of them a FBN1 mutation was proven. Patients with a bicuspid aortic valve or prior aortic surgery were excluded. Twenty-one healthy pediatric subjects (mean age 16.0 ± 2.6 years, 12 females) with a tricuspid aortic valve without history of cardiovascular disease and normal cardiac and aortic valve function, who underwent 4D flow CMR following a standard-of-care CMR examination, were collected from a retrospective chart review as approved by the IRB and were used as a healthy control group. The majority of these subjects underwent CMR for atypical chest pain after the inability of echocardiography to detect the origins of coronary arteries. The CMR revealed no morphological and functional cardiac or large vessel abnormalities in any of the healthy subjects. Written informed consent was obtained from all participants or their legal guardians for the addition of the 4D flow CMR to the standard-of-care CMR protocol.

### Magnetic resonance imaging

CMR examinations were performed on 1.5 T systems (MAGNETOM Avanto or Area, Siemens, Germany). All patients and healthy subjects underwent a standard-of-care CMR exam that included dynamic ECG gated two-dimensional (2D) cine steady state free precession (SSFP) imaging, for the evaluation of cardiac anatomy and function, as well as ECG gated and navigator triggered time-resolved contrast-enhanced MR angiography (CE-MRA) after administration of blood pool contrast media (gadofosveset trisodium, ABLAVAR, 0.03 mmol/kg, Lantheus, N. Billerica, MA), for evaluation of aortic morphology and dimensions. Sequence parameters for CE-MRA: spatial resolution: 1.3−1.5 × 1.3−1.5 × 1.3−1.7 mm^3^, echo time/repetition time: 1.2–1.6 ms/3.2–3.6 ms, flip angle: 18°. For assessment of aortic blood flow, 4D flow CMR was acquired during free breathing using respiratory navigator and prospective ECG-gating, with full volumetric coverage of the thoracic aorta in an oblique sagittal orientation. Standardized 4D flow sequence parameters were used throughout the study for age categories: 6–12 years and >12 years of age. Average 4D flow scan parameters were as follows: spatial resolution: 2.3−3.8 × 1.6−2.0 × 1.8−3.0 mm^3^, temporal resolution: 38.4–41.6 ms, echo time/repetition time: 2.4–2.7 ms/5.0–5.1 ms, flip angle: 15°, field of view: 250−320 × 141−250 mm^2^, matrix size: 160 × 70−130 and velocity sensitivity: 120–200 cm/s.

### Data analysis

#### Thoracic aortic size

**Fig. 1:**
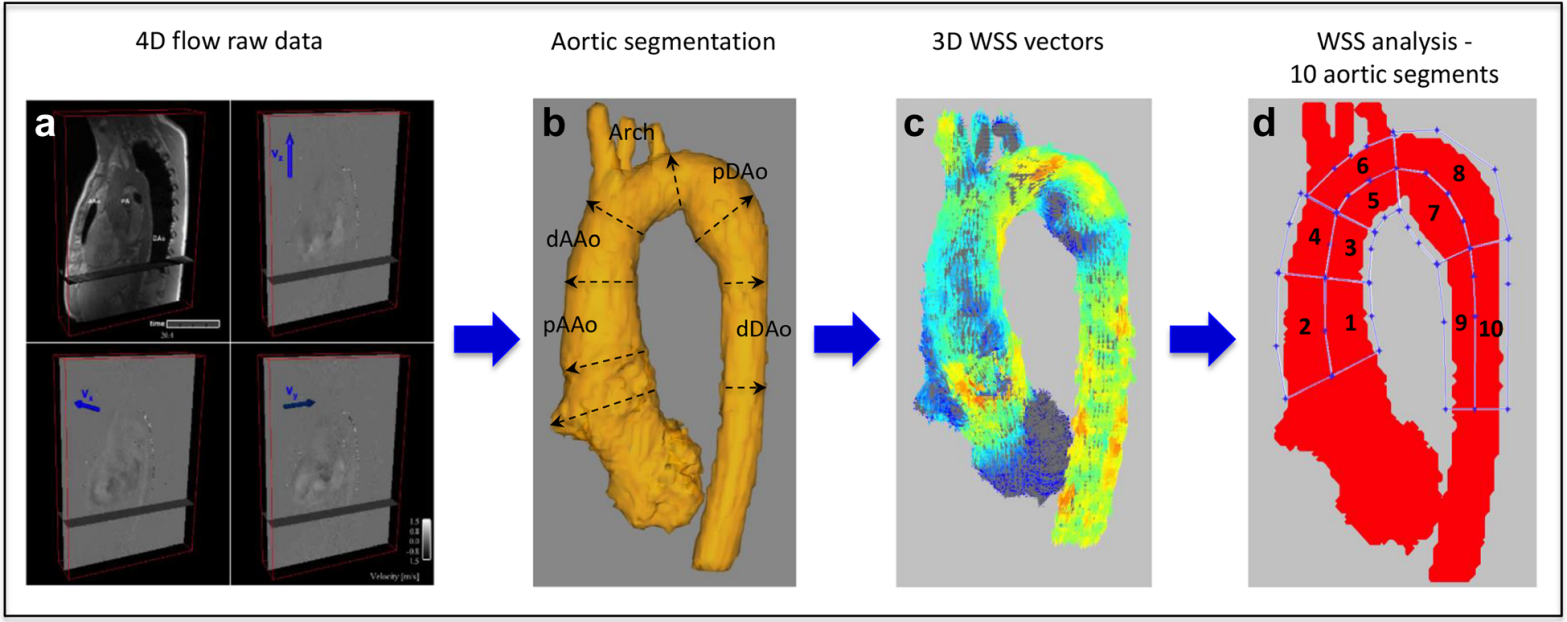
Aortic 4D flow data processing and analysis. **a** 4D flow raw data including anatomical and flow data. **b** volumetric aorta segmentation; levels of aortic diameter measurements. **c** calculation of systolic 3D WSS vector maps. **d** regional WSS analysis in ten aortic regions

Thoracic aortic diameters were determined from the CE-MRA exams using a 3D workstation with multiplanar reformatting capabilities (Vitrea, Vital Images, Minneapolis, MN). According to the international guidelines [17], maximum orthogonal aortic diameters were measured along the aorta from inner-edge to inner-edge at the following levels: STJ: sinotubular junction; midAAo: mid-ascending aorta, at the level of the right pulmonary artery; dAAo: distal ascending aorta, prior to innominate artery; distal aortic arch, between left carotid artery and left subclavian artery; aortic isthmus, at the level direct beyond the left subclavian artery; pDAo: proximal descending aorta, at the level of the ductal ampulla; midDAo: mid-descending aorta, at the level of the left atrium; dDAo: distal descending aorta, at the level of the diaphragm (Fig. 1b).

Maximum aortic root (Root) diameter was measured on a short-axis cine SSFP plane across the aortic root at peak systole [18, 19]. Change in aortic diameter along aortic segments was calculated and expressed as percentage change for the segments aortic root to midAAo and aortic isthmus to pDAo: $\left(\frac{\left| Aortic\  root- midAAo\right|}{Aortic\  root}\right)\ast 100\ \mathrm{and}\ \left(\frac{\left| Aortic\  isthmus- pDAo\right|}{Aortic\  isthmus}\right)\ast 100$. To account for the range of patient age and body size, aortic Z-scores were calculated for each patient from the CMR aortic measurements (AAo through pDAo) and the body surface area (BSA; determined according to the method of Mosteller) [20], using EchoIMS (Merge Healthcare, Chicago, IL). The Z-score is a nomogram-based metric for assessing aortic dilatation in pediatric patients in which a Z-score between −2 and +2 is considered normal [21]. While EchoIMS provides ultrasound derived normative data, there is currently no CMR-specific database, and as such, this method of normalization is the best available alternative and supported by previous studies [22, 23]. Particularly, Z-scores of the pDAo were calculated from the data on the aortic isthmus region of EchoIMS data. An aortic Z-score ≥ 2.0 indicates that the aortic diameter at that level is outside the upper normal range (≥ 2 standard deviations from the mean). Based on pDAo Z-scores, MFS patients were divided into subgroups for additional analysis: group 1: MFS Z-score pDAo ≥ 2.0; group 2: MFS Z-score pDAo < 2.0.

#### 4D flow data processing and 3D blood flow visualization

**Fig. 2:**
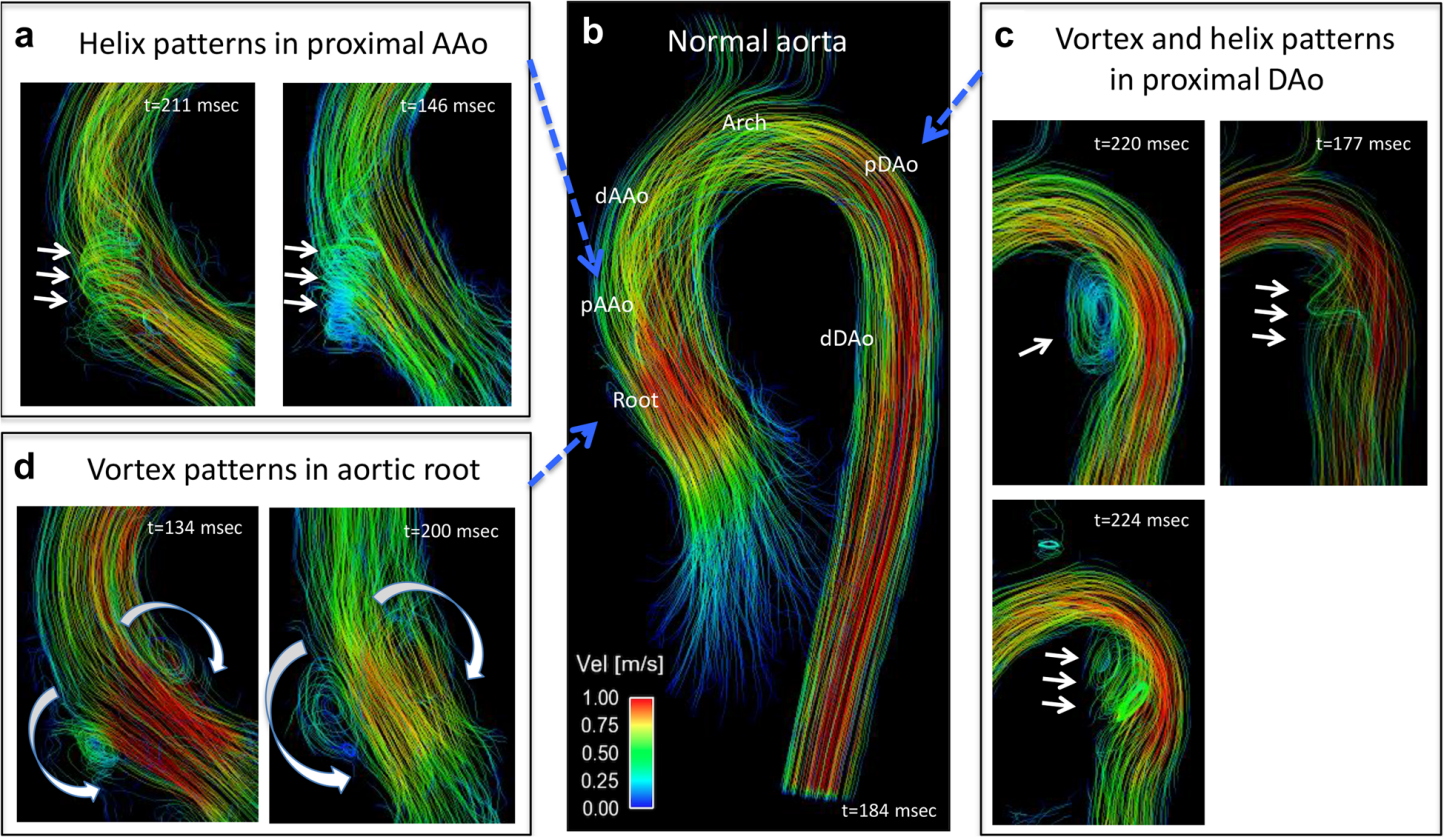
Aortic 3D blood flow characteristics (systolic streamlines). Panel **b** represents systolic streamline visualization of a normal aorta from a healthy control. Panels **a**, **c** and **d** illustrate typical secondary flow patterns in Marfan syndrome patients during systole, like a helix flow pattern from the aortic root into the pAAo (**a**, grade 2) and vortex and helix flow patterns in the pDAo (**c**, grade 2). **d** prominent vortex flow patterns in the aortic root

All 4D flow CMR data (Fig. 1a) were corrected for noise, velocity aliasing, Maxwell terms and eddy currents using Matlab-based in-house software (MathWorks, Natick, MA) as described previously [24]. A 3D phase-contrast MR angiogram was created from the corrected 4D flow CMR data and was used to semi-automatically define a 3D segmentation of the thoracic aorta (Mimics, Materialise, Leuven, Belgium) (Fig. 1b). The 3D segmentation was used to mask the velocity field for the generation of time-resolved 3D pathlines to visualize aortic 3D blood flow (Ensight, CEI, Apex, NC). Semiquantitative aortic flow pattern analysis using pathline movies was performed in MFS patients and healthy subjects in a blinded fashion and random order by two observers (CR and JR). A helical flow pattern was defined as rotational fluid motion around an axis parallel to the bulk fluid motion (i.e. along the longitudinal axis of the vessel). Vortical flow patterns were defined as revolving particles around a point within the vessel with a rotation direction deviating by >90° from the physiological flow direction. Vortex and helix flow pattern grading was assessed in three aortic segments (AAo, Arch and DAo) on a 3-point ordinal scale: 0: no vortex/helix patterns; 1: flow rotation <360°; 2: flow rotation >360° (Examples in Fig. 2).

#### Regional wall shear stress assessment

Based on the 3D segmentation, 3D mean systolic wall shear stress (WSS_mean_) along the entire aortic vessel wall was calculated from 4D flow velocity data (Fig. 1c) using a previously described algorithm, which was shown to provide good reproducibility [25, 26]. Briefly, WSS vector was estimated for each wall point on the aortic surface based on the 3D velocity spatial gradient perpendicular to the vessel wall. Systolic WSS vector magnitude was defined as the average over five cardiac time frames centered on the peak systolic time frame (i.e., peak systolic phase ± 2 phases). WSS_mean_ was calculated within ten aortic regions that were manually drawn based on anatomic landmarks (Fig. 1d): 1) inner and outer proximal ascending aorta (pAAo, from STJ to midAAo); 2) inner and outer distal ascending aorta (dAAo, from midAAo to the origin of the innominate artery); 3) inner and outer aortic arch (from origin innominate artery until left subclavian artery); 4) inner and outer proximal descending aorta (pDAo, beyond the left subclavian artery to mid-descending thoracic aorta); 5) inner and outer distal descending aorta (dDAo, from mid-descending thoracic aorta to the level of the aortic valve). Of note, the supra-aortic arch branches were excluded from the regional WSS measurements.

### Statistical analysis

Statistical analysis was performed using IBM SPSS Statistics 20.0 (SPSS Inc., Chicago, IL, USA). Baseline characteristics are provided as mean ± standard deviation for continuous variables and percentage for discrete variables. Shapiro-Wilk tests were performed to test for equal distribution of continuous variables. A chi-square test was used to investigate differences in gender distribution between the MFS and healthy subject groups. A non-paired t-test was performed to study differences in continuous variables from baseline characteristics between the two cohorts. For comparison of differences between aortic Z-scores as well as regional aortic WSS between the two cohorts a Mann Whitney test was used. A Kruskal-Wallis test was used to evaluate differences in aortic Z-score, size change and regional WSS between the two MFS subgroups, as well as between the MFS subgroup with a pDAo Z-score ≥ 2.0 and healthy subjects. The relationships between aortic dimensions and hemodynamic parameters were investigated using correlation analysis based on linear regression. A *p*-value < 0.05 was considered statistically significant for all statistical tests. Inter-observer agreement on qualitative assessment of helix and vortex flow formation between two observers was calculated using Cohen’s κ statistics. A κ value of 0.61–0.80 corresponded to substantial interobserver agreement; values of 0.41–0.60 corresponded to moderate agreement.

## Results

Patient characteristics are summarized in Table 1. As expected, MFS patients were typically taller than healthy subjects. All patients and healthy subjects had normal systolic biventricular function and no significant regurgitation of the mitral valve or aortic valve, except for one MFS patient who showed moderate mitral valve regurgitation.

**Table 1:** Baseline characteristics

	Marfan	Healthy controls	*p*-value
N (females)	25 (11)	21 (12)	0.375
Age (y)	15.6 ± 4.0	16.0 ± 2.6	0.720
Height (cm)	178.6 ± 17.3	161.2 ± 11.2	<0.001*
Weight (kg)	65.6 ± 20.2	58.8 ± 15.1	0.208
BSA (m^2^)	1.78 ± 0.36	1.61 ± 0.25	0.082
BMI (kg/m^2^)	20.1 ± 4.5	22.4 ± 4.0	0.081
LV ejection fraction (%)	55.7 ± 6.5	58.1 ± 3.7	0.243
Bilateral ectopia lentis (*n*, %)	5 (20%)	0	
Genetic mutation (*n*)			
FBN1 gene	22	-	
Unknown	3	-	
Cardiac medication, (*n,* %)	20 (80%)	0	
1. Beta-blocker	15 (60%)	-	
2. AT II-receptor antagonist	7 (28%)	-	
3. ACE inhibitor	3 (12%)	-	
4. Combination 1 + 2	4 (16%)	-	
5. Combination 1 + 3	1 (4%)	-	
Thoracic aortic size (Z-score)			
Aortic root	3.56 ± 1.45	0.49 ± 0.78	<0.001*
ST-junction	1.70 ± 1.28	−0.34 ± 0.77	<0.001*
Ascending aorta	0.21 ± 0.95	−0.54 ± 0.64	0.004*
Distal aortic arch	0.14 ± 0.90	−0.23 ± 0.66	0.155
Aortic isthmus	0.96 ± 0.94	0.43 ± 0.49	0.039*
Proximal descending aorta	2.02 ± 1.60	0.56 ± 0.66	<0.001*

The *p* value stems from non-paired t-test, for sex from Chi-square test and for thoracic aorta dimensions from the Mann Whitney test, *p < 0.05. *Abbreviations: ACE* angiotensin converting enzyme, *AT* angiotensin, *BMI* body mass index, *BSA* body surface area, *FBN1* fibrillin-1, *LV* left ventricular, *ST* sinotubular

### Thoracic aorta dimensions

As summarized in Table 1, the aortic diameter Z-scores were significantly greater in MFS patients compared to healthy subjects at all thoracic aorta levels (*p* ≤ 0.05), except for the distal aortic arch (*p* = 0.155). Aortic dilatation in the MFS population was most prominent in the aortic root and pDAo, with Z-scores of 3.56 ± 1.45 and 2.02 ± 1.60, respectively. Using a Z-score ≥ 2.0 to define aortic dilatation, *n* = 21 (84%) MFS patients had aortic root dilatation and *n* = 10 (40%) had dilatation of the STJ. Seven MFS patients (28%) had a pDAo Z-score ≥ 2.0.

### Aortic flow patterns

**Fig. 3:**
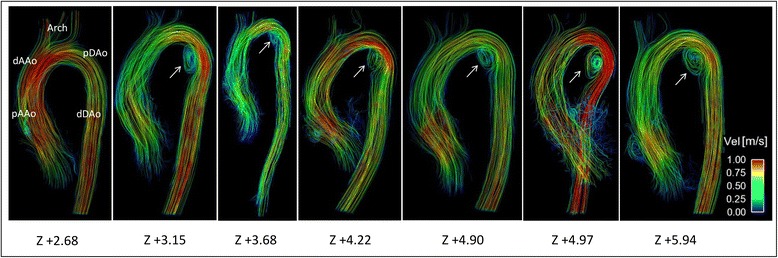
Aortic blood flow streamlines in MFS patients with Z-score ≥ 2.0 of the proximal descending aorta (*n* = 7). Local vortex flow patterns at the inner curvature of the pDAo (*arrows*) in six out of the seven MFS patients with dilated pDAo (Z-score ≥ 2.0)



There was moderate to substantial inter-observer agreement for grading of flow patterns within the AAo (Kappa 0.54) and DAo (Kappa 0.84) between the two observers. Examples of 3D blood flow patterns found in MFS patients are depicted in Fig. 2 (Panels a, c and d) and ﻿Additonal file 1: Video S1. Marked vortex flow patterns were noted in the pDAo region of 14 MFS patients and in only two healthy subjects (56% vs. 9.5% of total subjects, *p* = 0.002), with an average pDAo vortex grading significantly higher in MFS patients than healthy subjects (0.82 ± 0.83 vs. 0.14 ± 0.39, *p* < 0.001). These vortex flow patterns were most pronounced in MFS patients with a dilated pDAo (6 out of 7) (Fig. 3;﻿ Additional file 1: ﻿Video S1). Physiological vortex patterns in the sinuses of the aortic root were more often visible in MFS patients compared to healthy subjects (76.0% vs. 23.8%, *p* < 0.001; Fig. 2, Panel d; Additional files 1 and 2: Video S1 and S2). Non-physiological helical flow patterns with high strength (grade 2) originating from the aortic root were observed in 16% of the MFS patients and were not present in any of the healthy subjects (*p* < 0.05, Fig. 2, Panel a). Finally, throughout the AAo, no difference in presence or strength of major flow patterns was observed between the MFS patients and healthy subjects (MFS vs. healthy subjects: prevalence 76.0% vs. 57.1%, *p* = 0.17; average helix grading 1.18 ± 0.71 vs. 0.86 ± 0.76, *p* = 0.14). No helix or vortex flow patterns were present in the aortic arch for both MFS patients and healthy subjects.

**Additional file 1: Video S1.** Pathline visualization from a MFS patient’s aorta with local vortex flow pattern in the proximal descending aorta during systole and early diastole. Note the prominent vortex flow patterns in the sinuses of the aortic root. (MPG 3532 kb)




**Additional file 2: Video S2.** Pathline visualization of a normal aorta from a healthy subject. No local flow pattern abnormalities are present. (MPG 2044 kb)


### Segmental aortic WSS & correlations with diameter

**Fig. 4:**
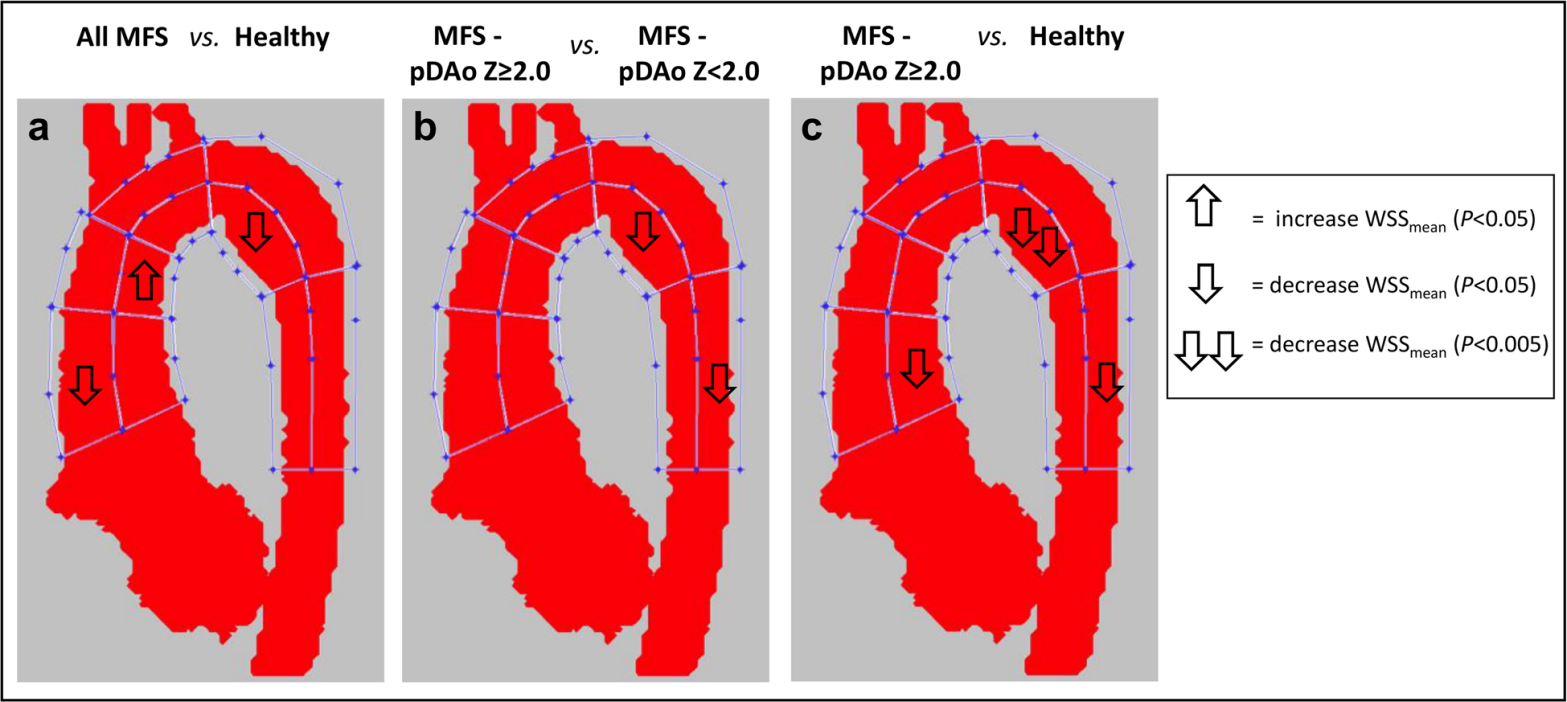
Comparison of mean systolic wall shear stress in the ten aortic regions between groups. **a** entire MFS cohort (*n* = 25) vs. healthy subjects (*n* = 21). **b** MFS with a pDAo Z-score ≥ 2.0 (*n* = 7) vs. MFS with a pDAo Z-score < 2.0 (*n* = 18). **c** MFS with pDAo Z-score ≥ 2.0 (*n* = 7) vs. healthy subjects (*n* = 21)

**Fig. 5:**
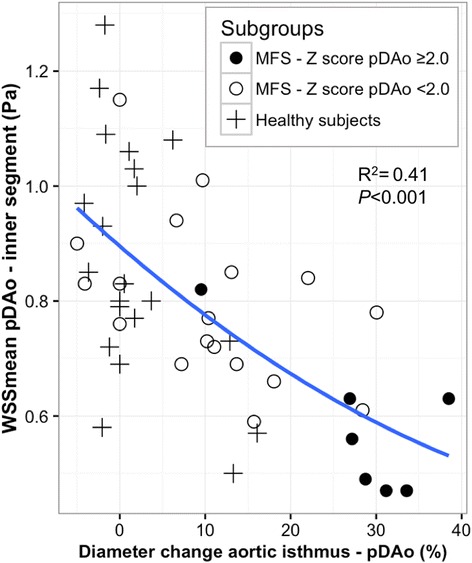
Relationship between size change and inner wall shear stress in the proximal descending aortic segment. Inverse relation between diameter change between aortic isthmus and pDAo segment (in % of isthmus diameter) and WSS_mean_ of the pDAo segment in MFS patients and healthy subjects

Figure 4a illustrates regional differences in WSS_mean_ between MFS patients and healthy subjects. MFS patients showed reduced WSS_mean_ in the pAAo outer segment (0.65 ± 0.12 vs. 0.73 ± 0.14 Pa, *p* = 0.029) and pDAo inner segment (0.74 ± 0.17 vs. 0.87 ± 0.21 Pa, *p* = 0.021) compared to healthy subjects (Fig. 4a). There was a higher WSS_mean_ in the inner segment of the dAAo in MFS patients (0.94 ± 0.14 vs. 0.84 ± 0.15 Pa, *p* = 0.036). Moreover, in the entire study cohort (MFS and healthy subjects), WSS_mean_ showed a strong inverse relationship with aortic dimensions, as well as with diameter changes along aortic segments. The most prominent association was found between the WSS_mean_ of the pDAo inner segment and both the pDAo Z-score (*R* = −0.53, *p* < 0.001) and the diameter change along the corresponding segment (*R* = −0.64, *p* < 0.001; Fig. 5).

### Subgroup analysis according to proximal DAo dilatation

Based on pDAo Z-scores, MFS patients were divided into subgroups for additional analysis. Seven MFS patients had a Z-score pDAo ≥ 2.0 (group 1), whereas 18 MFS patients had a Z-score pDAo < 2.0 (group 2). Age, gender and BSA were comparable between the MFS subgroups with dilated pDAo (Z-score ≥ 2.0) and healthy subjects (Table 2). Differences in aortic diameter and segmental WSS between healthy subjects and MFS subgroups as well as between the two MFS subgroups are summarized in Table 2. Dimensions of the entire thoracic aorta in the MFS subgroup with dilated pDAo showed significantly greater Z-scores compared to both other groups, except for the aortic root (Table 2). MFS patients with a dilated pDAo showed significantly lower WSS_mean_ in the inner pDAo aortic segment (*p* < 0.05) compared to both the MFS patients with pDAo Z-score < 2.0 and the group of healthy subjects (Fig. 4b-c and Table 2). Furthermore, MFS patients with dilated pDAo also showed lower WSS_mean_ at the inner pAAo segment (*p* < 0.05) and outer dDAo segment (*p* < 0.05) compared to healthy subjects.

**Table 2:** Comparison of aortic dimension and WSS between MFS groups and healthy subjects

	Group 1 MFS - pDAo Z ≥ 2.0 (*n* = 7)	Group 2 MFS - pDAo Z < 2.0 (*n* = 18)	Group 3 Healthy subjects (*n* = 21)
Age (y)	15.6 ± 4.5	15.6 ± 4.0	16.0 ± 2.6
Females (*n*)	3	8	12
BSA (m^2^)	1.6 ± 0.4	1.8 ± 0.3	1.6 ± 0.3
Thoracic aortic size (Z-score)			
Z-score Aortic Root**	4.4 ± 1.3	3.2 ± 1.4	0.5 ± 0.8
Z-score STJ**	3.0 ± 0.9	1.2 ± 1.0	−0.3 ± 0.8
Z-score AAo**	1.1 ± 0.7	−0.1 ± 0.8	−0.5 ± 0.6
Z-score distal Arch*	0.8 ± 0.7	−0.1 ± 0.9	−0.2 ± 0.7
Z-score Isthmus**	2.0 ± 0.7	0.6 ± 0.7	0.4 ± 0.5
Z-score proximal DAo**	4.2 ± 1.1	1.2 ± 0.6	0.6 ± 0.7
Δ diameter Root-AAo (%)**	30.9 ± 4.0	33.8 ± 7.0	19.0 ± 7.0
Δ diameter Isthmus-pDAo (%)**	27.9 ± 9.1	10.4 ± 10.1	1.9 ± 5.7
WSS_mean_ (Pa)			
1. Inner pAAo*	0.69 ± 0.18	0.81 ± 0.09	0.86 ± 0.16
2. Outer pAAo	0.59 ± 0.11	0.67 ± 0.11	0.73 ± 0.14
3. Inner dAAo*	0.85 ± 0.18	0.97 ± 0.11	0.85 ± 0.15
4. Outer dAAo	0.75 ± 0.14	0.87 ± 0.11	0.83 ± 0.14
5. Inner Arch	0.83 ± 0.13	0.99 ± 0.13	0.91 ± 0.16
6. Outer Arch	0.71 ± 0.09	0.80 ± 0.11	0.79 ± 0.14
7. Inner pDAo*	0.58 ± 0.13	0.80 ± 0.14	0.87 ± 0.21
8. Outer pDAo	0.76 ± 0.13	0.89 ± 0.10	0.86 ± 0.12
9. Inner dDAo	0.75 ± 0.09	0.89 ± 0.16	0.89 ± 0.17
10. Outer dDAo*	0.78 ± 0.05	0.99 ± 0.18	0.97 ± 0.16

Significant across subgroups using a Kruskal-Wallis test. *p < 0.05, **p < 0.001. *Abbreviations: pAAo* proximal ascending aorta, *dAAo* distal ascending aorta, *Arch* aortic arch, *pDAo* proximal descending aorta, *dDAo* distal descending aorta

## Discussion

The findings of this study demonstrate that pediatric MFS patients have significantly lower WSS_mean_ in the pAAo and pDAo than age-matched healthy controls. These regions in the aorta correspond to the locations where aortic dissection and aortic rupture often originate [2, 4, 5]. The subgroup of MFS patients with already dilated pDAo showed more prominent local vortex flow patterns and markedly lower WSS_mean_ in the dilated pDAo region, when compared to non-dilated pDAo MFS patients and healthy controls. Furthermore, an inverse relationship between regional aortic diameter and WSS_mean_ was observed in the entire study group.

Aortic dilatation in MFS patients may start in early childhood, warranting close clinical follow up of the diameters in the entire thoracic aorta from the time of diagnosis. The average aortic size and the heterogeneous involvement of the pDAo regarding dilatation found in this study are in concordance with previous studies [27, 28]. Although MFS is known to be prone to ascending aortic complications, a substantial part of the aortic dissections in MFS occur in the pDAo before reaching surgical cut-off levels. European studies reported the pDAo to be the site of the first aortic complications in 16–18% of the MFS patients [2, 29]. This proportion will increase after elective surgery of the AAo [4, 5]. Changes in WSS in the aorta caused by changes in flow may be the key to better understand aortic disease development in MFS.

### Aortic flow patterns and wall shear stress

Mechanisms leading to progressive aortic dilatation and elongation are not fully understood. Recently, aortic flow and derived hemodynamic factors from CMR, including WSS, have been associated with focal evidence of molecular and architectural tissue dysfunction in patients with bicuspid aortic valve and aortopathy [15]. This study confirms that children and adolescent MFS patients already have disturbed aortic flow patterns in the thoracic aorta. The regions of prominent flow alterations were the aortic root and pDAo, with a significantly higher prevalence and strength of vortex flow patterns found in the pDAo in MFS compared to healthy subjects. Although it is known that small vortex flow patterns in the pDAo sometimes occur in normal aortas with an aortic spindle (i.e. a normal variation in the aortic contour not to be confused with an aneurysm) [30], the high prevalence and extensive nature found in these young MFS patients in this study is remarkable and consistent with observations by two previous CMR studies in MFS, with prevalence rates ranging from 54 to 70% [8, 31]. Local aortic geometry seems to be related to the formation of these flow phenomena as the MFS subjects with most dilated pDAo segments (Z-score ≥ 2.0) showed the most extensive focal vortex flow patterns in this study.

Several studies previously demonstrated that visibly pathologic flow patterns could lead to measurable alterations in WSS [32, 33]. Interestingly, the observed vortex flow patterns in the pDAo in MFS patients in this study corresponded to the reduced WSS_mean_ observed in the same segment. A similar relationship has been described in a longitudinal follow-up study of MFS patients after root replacement where vortex flow disturbances within the pDAo segment together with reduced local WSS measures developed [34]. In addition to the lower WSS_mean_ in the pDAo segment, considerable differences in WSS_mean_ were observed in the pAAo outer segment of the MFS patients compared to healthy subjects. However, no obvious flow pattern differences in the AAo were detected between MFS patients and the controls, except for local helix formation originating from the aortic root into the proximal AAo in four MFS patients. The significant difference in AAo size between the MFS and healthy controls could have contributed to this reduced regional WSS_mean_.

Few studies have investigated aortic WSS in MFS disease cohorts so far [9, 10] and comparable investigations on aortic WSS were conducted in similar patients groups with dilative aortopathy of unknown origin (i.e. non-bicuspid valve related diseases) [32, 35]. As in MFS patients, these patients have in common that the AAo is more or less dilated and these studies revealed similar aortic WSS results. In general, lower systolic WSS values were found along the dilated thoracic aorta in both these patient groups, as provided by 2D planar WSS quantification methods [9, 32, 35]. Similar to our findings, the specific locations of reduced systolic WSS were found at the outer curvature of the pAAo in most of these studies [9, 35]. Contrary to our results, one of the WSS studies in pediatric MFS patients showed inhomogeneous but relatively higher peak systolic WSS values at focal points on the vessel lumen circumference in the AAo compared to a healthy control group [10]. Although this is inconsistent to our findings, the higher WSS found in that study might be explained by the fact that the MFS patients were younger and had similar or even significantly smaller indexed aortic dimensions compared to the older healthy control group. Indeed, both factors (i.e. younger age and smaller vessel dimensions) have been proven to be associated with relatively higher WSS measures [36, 37]. The pDAo WSS in the MFS cohorts from these 4D flow CMR studies was equal or lower in MFS, but statistically similar compared to the WSS of healthy controls [9, 10]. It should be noted that the aortic WSS measurements in these comparative studies were all based on 2D planar WSS quantification methods, which means that WSS measurements are derived from single 2D cross-sectional planes along the thoracic aorta.

In this study, we used a novel approach providing 3D WSS along the entire aortic wall that was recently developed [25, 26]. The major advantage of this 3D WSS approach is that the entire aorta is included rather than sampling only a fraction of the vessel wall, thus reducing the chance of missing important regional variations in WSS. Moreover, it allows regional comparison to control references and no manual placement of 2D cross-sectional planes is needed, which is more subject to observer variability. In addition, the estimation of 3D WSS over a certain regional vessel surface enables to study relationships between regional hemodynamics and geometric measures, for instance aortic diameter or diameter changes between vessel segments.

### Aortic WSS and diameter

The findings of our study revealed a close interaction between altered aortic diameter and regional WSS_mean_ in MFS patients and healthy subjects. First, an inverse relationship between WSS_mean_ and aortic diameter was observed, which is congruent with existing literature [35, 36, 38]. The most obvious correlation was found for the pDAo segment, where both diameter and diameter change between the aortic isthmus and the largest pDAo dimension were inversely proportional to WSS_mean_. Furthermore, subgroup analysis of MFS patients with a dilated pDAo showed the lowest WSS_mean_ in the corresponding region. Second, the dilation of the aortic root and STJ in MFS patients may have resulted in reduced WSS_mean_ values in the pAAo segment and might explain the WSS_mean_ asymmetry between the inner and outer pAAo segments. Such WSS asymmetry was also observed in the pAAo segment in another 4D flow CMR study on MFS patients [9]. Moreover, an *in-vitro* computational fluid dynamic (CFD) investigation about the effect of morphological changes in the aortic root on flow hemodynamics supports these findings. That CFD study, which was conducted with MFS-specific aortic root models of increasing size, showed that progressive root dilatation resulted in a different orientation of the blood stream jet in the aortic root and pAAo [39]. It is conceivable that a change in flow displacement from the sinus side towards the commissure side has resulted in the altered distribution of WSS_mean_ that was observed in the pAAo in the MFS patients of this study.

### Clinical perspectives

Current management strategies for preventive aortic replacement in MFS are based on aortic diameters or rate of dilatation. However, this strategy remains imperfect and has led to a search for additional markers of disease severity and predictors of adverse vascular events, such as hemodynamic parameters. The results of this study showed already hemodynamic differences in young MFS patients compared to age-appropriate healthy subjects and determined close relationships between regional aortic geometry, flow patterns and 3D WSS over certain vessel regions. This relationship was most evident for the pDAo. Whether these hemodynamic parameters (i.e. flow and WSS) may add in the prediction of progressive dilatation, adverse events or optimization of management strategies in future in addition to the current diameter measurements is unknown and requires further longitudinal follow-up. In this regard and in line with our findings, a recent longitudinal 4D flow follow-up study on MFS patients after preventive valve-sparing aortic root replacement reported the occurrence of helical and vortex flow pattern formation along the inner curvature of the pDAo in 50% of the patients [34]. Additionally, in that report, a single case was described with newly derived regional vortex formation in the pDAo and altered WSS in that region prior to the development of an intramural hematoma with subsequent aortic dissection type B in the pDAo. This occurred 5 years after root replacement and the changes in flow and WSS parameters are noteworthy and suggest that these factors may play a role in the onset of the adverse event [34]. Together with our results, these observations show the clinical potential and future perspectives of comprehensive aortic flow hemodynamics assessments and monitoring of longitudinal changes by 4D flow imaging for optimization of preventive decision making. Based on these and our data, one might speculate that at least the MFS patients with pDAo Z-score ≥ 2.0, abnormal vortex flow patterns and decreased local WSS_mean_, are at higher risk for adverse cardiovascular events. Additional longitudinal studies are warranted to further elucidate the diagnostic value of inter-individual differences in MFS hemodynamics and the applicability of 4D flow parameters in prediction models.

### Study limitations

The current study is subject to several limitations. In this observational study there might be some selection bias. Although MFS patients underwent CMR as a regular part of their lifelong clinical follow-up according to the MFS imaging guidelines, we cannot completely exclude the possibility that some of them received MR imaging based on previously abnormal findings on echocardiographic imaging. These results therefore may not be generalizable. However, it is known that the MFS patients individually can show a large heterogeneity in the involvement of heart and cardiovascular problems, e.g. the onset and the progression of disease over time.

To account for patient age and body size for aortic dimensions comparison in children, aortic Z-scores were calculated using EchoIMS. While EchoIMS provides ultrasound-derived normative data, there is currently no large pediatric CMR-specific database and this method of normalization is the best available alternative, is used in clinical practice and has been applied in previous CMR studies [22, 23].

Finally, potential sources of error, including the limited spatial resolution of 4D flow CMR, could have resulted in underestimation of 3D WSS_mean_ measurements. However, as both patient and healthy control groups underwent MR imaging at the same scanners with similar scan parameters, relative differences in WSS_mean_ between the two groups remain useful. Furthermore, 3D segmentation of the aorta based on the time-averaged systolic portion of the cardiac cycle is a potential source of error in this 4D flow study. However, averaging WSS over five systolic phases of the cardiac cycle with our algorithm, and using a well-defined segmentation protocol have been proven to produce low variability [26].

## Conclusions

MFS children and young adults have altered aortic flow patterns and differences in WSS that were most pronounced in the pAAo and pDAo, which correspond to the locations where aortic dissection and aortic rupture often originate in these patients. Furthermore, close relationships between the regional aortic size and the presence of abnormal flow patterns and WSS were demonstrated and were more evident in the pDAo segment. These findings indicate that hemodynamic parameters may be discriminative and potentially valuable additional markers of disease severity. Additional longitudinal studies correlating changes in hemodynamic parameters (aortic flow profiles and WSS) with aortopathy are needed to establish the role of these parameters in disease progression and risk prediction of adverse events in MFS patients.

## Abbreviations

2D: Two-dimensional
3D: Three-dimensional
4D flow CMR: Three-dimensional phase-contrast CMR with three-directional velocity encoding
AAo: Ascending aorta
CE-MRA: Contrast-enhanced MR angiography
CFD: Computational fluid dynamic
CMR: Cardiovascular magnetic resonance
dAAo: Distal ascending aorta
DAo: Descending aorta
dDAo: Distal descending aorta
FBN1: Fibrillin-1
IRB: Institutional Review Board
MFS: Marfan syndrome
midAAo: Mid-ascending aorta
midDAo: Mid-descending aorta
pAAo: Proximal ascending aorta
pDAo: Proximal descending aorta
SSFP: Steady state free precession
STJ: Sinotubular junction
Vel: Velocity
WSS: Wall shear stress
WSS_mean_: 3D mean systolic wall shear stress
